# Sarilumab and adalimumab differential effects on bone remodelling and cardiovascular risk biomarkers, and predictions of treatment outcomes

**DOI:** 10.1186/s13075-020-02163-6

**Published:** 2020-04-07

**Authors:** Cem Gabay, Gerd R. Burmester, Vibeke Strand, Jérôme Msihid, Moshe Zilberstein, Toshio Kimura, Hubert van Hoogstraten, Susan H. Boklage, Jonathan Sadeh, Neil M. H. Graham, Anita Boyapati

**Affiliations:** 1 University Hospitals of Geneva, Geneva, Switzerland; 2 Charité - University Medicine Berlin, Berlin, Germany; 3 Stanford University, Palo Alto, CA USA; 4 Sanofi, Chilly-Mazarin, France; 5 Sanofi Genzyme, Bridgewater, NJ USA; 6 Regeneron Pharmaceuticals Inc., Tarrytown, NY USA

**Keywords:** Rheumatoid arthritis, Sarilumab, Biomarkers, Biologic disease-modifying antirheumatic drug, Cardiovascular risk, Bone remodelling, Acute-phase response, Synovial inflammation

## Abstract

**Background:**

Interleukin-6 (IL-6) is a pleiotropic cytokine that plays a key role in the pathogenesis of rheumatoid arthritis. Sarilumab is a human monoclonal antibody that binds membrane-bound and soluble IL-6 receptor-α to inhibit IL-6 signalling. The aim of this study was to compare the effects of sarilumab and adalimumab (a tumour necrosis factor alpha inhibitor) monotherapy on levels of circulating biomarkers associated with the acute-phase response, bone remodelling, atherothrombosis, anaemia of chronic disease and markers purported to reflect synovial lymphoid and myeloid cell infiltrates, as well as the potential of these biomarkers to differentially predict clinical and patient-reported outcomes with sarilumab vs. adalimumab.

**Methods:**

In this post hoc analysis, serum samples were analysed at baseline and prespecified post-treatment timepoints up to week 24 in adults with moderate-to-severe active rheumatoid arthritis intolerant of or inadequate responders to methotrexate from the MONARCH trial (NCT02332590).

**Results:**

Greater reductions in C-reactive protein (CRP; − 94.0% vs. –24.0%), serum amyloid A (SAA; − 83.2% vs. –17.4%), total receptor activator of nuclear factor-κB ligand (RANKL; − 18.3% vs. 10.5%) and lipoprotein (a) (− 41.0% vs. –2.8%) were observed at week 24 with sarilumab vs. adalimumab, respectively (adjusted *p* < 0.0001). Greater increases in procollagen type 1 *N*-terminal propeptide (P1NP) were observed with sarilumab vs. adalimumab at week 24 (22.8% vs. 6.2%, *p* = 0.027). Patients with high baseline SAA, CRP and matrix metalloproteinase-3 (MMP-3) were more likely to achieve clinical efficacy, including American College of Rheumatology 20% improvement criteria and Disease Activity Score (28 joints)-CRP < 3.2, and report improvements in patient-reported outcomes, including Health Assessment Questionnaire-Disability Index and pain visual analogue scale, with sarilumab than adalimumab.

**Conclusion:**

Sarilumab was associated with greater positive effects on bone remodelling and decreases in biomarkers of the acute-phase response, synovial inflammation and cardiovascular risk vs. adalimumab. High baseline concentrations of SAA, CRP and MMP-3 are predictive of clinical and patient-reported outcome responses to sarilumab treatment and prospective validation is warranted to confirm these results.

**Trial registration:**

ClinicalTrials.gov, NCT02332590. Registered on 5 January 2015

## Background

Patients with rheumatoid arthritis (RA) develop bone and cartilage damage in synovial joints as a result of chronic inflammation, which is mediated by pro-inflammatory cytokines such as interleukin-6 (IL-6) and tumour necrosis factor alpha (TNF-α) [1–3]. In RA, elevated circulating cytokine concentrations trigger bone and cartilage destruction through activation of signalling cascades that lead to the stimulation of osteoclasts via bone-resorptive factors (e.g. receptor activator of nuclear factor-κB ligand [RANKL]) and joint-destructive proteins (e.g. matrix metalloproteinases) [3, 4]. Underlying joint damage leads to long-term impairments in physical function [5].

IL-6 is a pleiotropic cytokine that plays a role in inflammatory, metabolic, neural and regenerative processes [6]. IL-6 operates through two distinct mechanisms—classic (*cis*) and *trans*-signalling—which expands the range of its actions and contributes towards the systemic manifestations and co-morbidities commonly associated with RA, including the acute-phase response, osteoporosis, fatigue, depression, anaemia and cardiovascular (CV) disease [7–9]. Patients with RA have an increased risk of CV events, including myocardial infarction and stroke, relative to healthy individuals [10]. It is understood that pro-inflammatory cytokines promote endothelial dysfunction and structural vessel abnormalities and induce other CV risk factors, including changes in lipid levels, insulin resistance and oxidative stress [11]. In addition, significantly elevated levels of lipoprotein (a) (Lp [a]), a biomarker of CV risk which is involved in both inflammation and thrombosis, have been observed in patients with RA compared with healthy controls [12].

Sarilumab is a human monoclonal antibody that binds membrane-bound and soluble IL-6 receptor-α to inhibit IL-6 signalling. It is approved for the treatment of adults with moderate-to-severe active RA as monotherapy and in combination with conventional synthetic disease-modifying anti-rheumatic drugs (csDMARDs) [13–15]. Adalimumab is a human monoclonal antibody that blocks TNF-α and is approved for the treatment of RA, among other conditions [16]. The efficacy of sarilumab vs. adalimumab was evaluated in the MONARCH phase III randomized controlled trial (NCT02332590) [15]. Sarilumab monotherapy was superior to adalimumab monotherapy, as demonstrated by greater reduction in Disease Activity Score (28 joints) using erythrocyte sedimentation rate (DAS28-ESR) in adults with moderate-to-severe active RA who were intolerant of or inadequate responders to methotrexate (MTX-INT/MTX-IR) [15, 17]. The safety profiles of both therapies were consistent with anticipated class effects.

In this post hoc analysis, levels of circulating biomarkers were evaluated at baseline and after treatment, which were associated with: (1) the acute-phase response (C-reactive protein [CRP] and serum amyloid A [SAA]), (2) bone remodelling (procollagen type 1 *N*-terminal propeptide [P1NP], osteocalcin [OC], total RANKL and osteoprotegerin [OPG]), (3) synovial inflammation (matrix metalloproteinase-3 [MMP-3]), (4) purported to reflect synovial lymphoid (chemokine [C-X-C motif] ligand 13 [CXCL13]) and myeloid cell (soluble intercellular adhesion molecule-1 [sICAM-1]) infiltrates, (5) atherothrombosis (Lp [a]) and (6) anaemia of chronic disease (hepcidin, ferritin, total iron-binding capacity [TIBC] and iron). The effects of sarilumab and adalimumab monotherapy on the levels of these markers were examined, as well as the potential of these markers at baseline to differentially predict the efficacy of or improvement in patient-reported outcomes (PROs) with sarilumab vs. adalimumab. This analysis did not evaluate the relationship between biomarkers and safety parameters.

## Methods

This phase III active-comparator randomized controlled trial has been described in full previously [15]. In brief, MTX-INT/IR patients were randomized to sarilumab 200 mg every 2 weeks (q2w) or adalimumab 40 mg q2w for 24 weeks. At week 16, dose escalation to weekly adalimumab was permitted for those who did not achieve ≥ 20% improvement in tender and swollen joint counts. The trial was conducted in accordance with Good Clinical Practice and with the principles of the Declaration of Helsinki; all protocols and patient information materials were approved by appropriate ethical review boards and all patients provided written informed consent.

### Efficacy and PRO endpoints

Efficacy endpoints included the following: proportion of patients achieving ≥ 20/50/70% improvement according to American College of Rheumatology criteria (ACR20/50/70), Clinical Disease Activity Index (CDAI) ≤ 2.8, CDAI ≤ 10, DAS28 using CRP (DAS28-CRP) or DAS28-ESR < 2.6 and DAS28-CRP or DAS28-ESR < 3.2.

PROs evaluated in the study were previously described for the overall intent-to-treat (ITT) population [17] and, evaluated as change from baseline at week 24, included Patient Global Assessment of disease activity visual analogue scale (VAS), Health Assessment Questionnaire-Disability Index (HAQ-DI), pain VAS, Functional Assessment of Chronic Illness Therapy (FACIT)-Fatigue, morning stiffness VAS, rheumatoid arthritis impact of disease (RAID) score and Medical Outcomes Study Short-Form (36-item) Health Survey (SF-36) physical (PCS) and mental (MCS) component summary scores, which include the physical functioning, role-physical, bodily pain, general health, vitality, social functioning, role-emotional and mental health domains.

### Serum collection and biomarker analysis

Patients were selected for this biomarker analysis if they had been randomized and treated with sarilumab or adalimumab during the double-blind period and had provided written informed consent for future use of samples, with a serum sample drawn pre-dose (baseline) and evaluable (biomarker population). Serum samples were collected and stored frozen at baseline and post-treatment through week 24 from 307 patients in the ITT population (sarilumab, *n* = 153; adalimumab, *n* = 154).

Biomarkers were analysed retrospectively (except CRP) at one or two post-baseline timepoints through week 24 (Table S1). Timepoints selected for analysis were based on either previous data following sarilumab treatment [18, 19] or on literature suggesting either acute or latent effects of RA therapy on specific markers. The assay characteristics for most biomarkers have been described previously [18].

### Statistical analysis

Baseline biomarker levels were compared between treatment groups using a Wilcoxon test. Spearman’s ranked correlations at baseline were computed in the overall biomarker population.

To evaluate pharmacodynamic changes in circulating biomarker concentrations between treatment groups at each timepoint, absolute and percentage changes from baseline were described. In addition, the percentage changes in biomarker concentrations were analysed using non-parametric methods because of non-normal distributions. For biomarkers measured once post-baseline, a rank-based analysis of covariance (ANCOVA) adjusted on baseline value was implemented. For biomarkers measured twice post-baseline, a mixed-effect model with repeated measures was performed on rank-transformed data (analysis of variance [ANOVA]-type method), with treatment, visit and treatment-by-visit interaction as fixed effects, baseline biomarker value transformed in rank, and baseline biomarker value transformed in rank-by-visit interaction as fixed covariates, assuming an unstructured covariance structure. The log-transformed RANKL/OPG ratio was analysed using a mixed model for repeated measures with response, visit and response-by-visit interaction as fixed effects, baseline biomarker value and baseline biomarker value-by-visit interaction as fixed covariates, and assuming an unstructured covariance structure separately by treatment group. *p* values were adjusted for false discovery rate (Benjamini–Hochberg 5% threshold). The number of patients with abnormal biomarker levels at baseline (according to the reference ranges provided by the testing laboratory) that normalized with treatment was compared between groups using a *χ*^2^ test; nominal *p* values are reported.

Subgroup analyses were performed according to the use of systemic steroids at baseline. Percentage changes from baseline in biomarker levels were analysed separately in each subgroup, and nominal *p* values were provided.

Percentage changes in biomarker concentrations at week 24 were compared between clinical responders and non-responders at the same visit within each treatment group using similar non-parametric methods. *p* values were also adjusted for false discovery rate.

For binary efficacy endpoints, predictive effects of baseline biomarker values on sarilumab efficacy vs. adalimumab were tested using a logistic regression with treatment group and region as fixed effects, baseline biomarker value as a continuous covariate and the baseline biomarker-by-treatment group interaction. For continuous PROs, a linear regression was used with the same effects as above, as well as the baseline PRO value as a covariate. Nominal values for the interaction are reported to assess the predictive value of the biomarkers. Similar analyses were performed after categorization of patients into high, medium and low biomarker levels at baseline using tertile values in the biomarker population. In addition, pairwise comparisons of responses between sarilumab and adalimumab were performed separately in patients with high, medium and low biomarker levels, and the Mantel–Haenszel estimates of odds ratios (ORs), stratified by region, and 95% confidence intervals (CIs) were derived and graphically represented using forest plots. For continuous PROs, a linear regression was performed separately in each biomarker tertile and differences in least squares mean (LSM) changes with 95% CI between both treatments were provided.

Differential combinations of circulating CXCL13 and sICAM-1 (low or high levels defined relative to baseline median levels) were assessed for prediction of response to sarilumab, using Mantel–Haenszel estimates of ORs derived for each combination.

All analyses were performed using SAS version 9.2 or higher (SAS Institute Inc., Cary, NC, USA).

## Results

### Baseline demographics, disease characteristics, efficacy and biomarker levels

Baseline demographics and disease characteristics of the biomarker population were generally similar to the overall ITT population (Table 1). Overall, efficacy and PROs were also generally similar between the ITT and biomarker populations (Table S2).

**Table 1:** Baseline patient demographics and disease characteristics

	ITT population		Biomarker population	
	Adalimumab 40 mg q2w (*n* = 185)	Sarilumab 200 mg q2w (*n* = 184)	Adalimumab 40 mg q2w (*n* = 154)	Sarilumab 200 mg q2w (*n* = 153)
Age, years	53.6 (11.9)	50.9 (12.6)	53.3 (12.0)	50.4 (12.5)
Sex, female, %	81.1	85.3	78.6	83.7
Duration of RA, years	6.6 (7.8)	8.1 (8.1)	6.6 (8.1)	7.9 (8.1)
ACPA positive, %	76.7	75.3	76.2	73.8
RF positive, %	64.8	66.9	63.8	66.0
Treated with 1/2/≥ 3 prior csDMARDs/immunosuppressive agents, %	47.6/31.4/21.1	45.1/31.0/23.9	48.1/31.8/20.1	46.4/32.0/21.6
Oral corticosteroid use, %	56.2	53.3	57.1	51.6
Tender joint count	26.7 (13.6)	28.0 (13.2)	26.9 (13.9)	28.1 (13.4)
Swollen joint count	17.5 (10.3)	18.6 (10.7)	17.3 (10.1)	18.5 (10.6)
HAQ-DI	1.6 (0.6)	1.6 (0.6)	1.6 (0.6)	1.6 (0.6)
CRP, mg/L	24.1 (31.0)	17.4 (21.3)	23.6 (31.1)	17.4 (21.9)
DAS28-ESR	6.8 (0.8)	6.8 (0.8)	6.8 (0.8)	6.8 (0.8)
DAS28-CRP	6.0 (0.9)	6.0 (0.9)	6.0 (0.9)	6.0 (0.9)
CDAI	42.4 (12.0)	43.6 (12.1)	42.8 (11.9)	43.8 (12.0)
Pain VAS (0–100 mm)	71.4 (19.0)	71.6 (18.7)	71.3 (18.6)	71.2 (19.1)
Patient global VAS (0–100 mm)	67.8 (18.4)	68.0 (17.5)	68.6 (18.2)	67.8 (17.9)
SF-36 PCS	31.4 (6.6)	30.7 (6.2)	31.5 (6.5)	30.4 (6.2)
SF-36 MCS	37.1 (11.8)	36.7 (10.7)	37.4 (12.4)	37.0 (11.2)
Morning stiffness VAS (0–100 mm)	68.0 (21.4)	70.8 (19.0)	68.2 (21.7)	70.6 (18.8)
FACIT-fatigue (0–52)	24.0 (10.3)	24.0 (9.0)	23.9 (10.4)	23.4 (9.1)
Physician global VAS (0–100 mm)	66.0 (17.1)	66.3 (15.7)	66.2 (16.4)	67.3 (14.9)
RAID (0–10)	6.4 (2.0)	6.7 (1.7)	6.4 (2.1)	6.6 (1.7)

Mean (standard deviation) unless otherwise stated

*ACPA* anti-citrullinated protein antibody, *CDAI* Clinical Disease Activity Index, *CRP* C-reactive protein, *csDMARD* conventional synthetic disease-modifying anti-rheumatic drug, *DAS28-CRP* Disease Activity Score (28 joints) using C-reactive protein, *DAS28-ESR* Disease Activity Score (28 joints) using erythrocyte sedimentation rate, *FACIT* Functional Assessment of Chronic Illness Therapy, *HAQ-DI* Health Assessment Questionnaire-Disability Index, *ITT* intent-to-treat, *MCS* mental component summary, *PCS* physical component summary, *q2w* every 2 weeks, *RA* rheumatoid arthritis, *RAID* rheumatoid arthritis impact of disease, *RF* rheumatoid factor, *SF-36* Medical Outcomes Study Short-Form (36-item) Health Survey, *VAS* visual analogue scale

Baseline serum levels of most biomarkers were similar between treatment groups, except for Lp(a), which was higher in the adalimumab than the sarilumab groups (Lp [a]: median 235.5 vs. 179.0 mg/L, respectively; Wilcoxon test *p* value 0.039; Table S3).

**Fig. 1:**
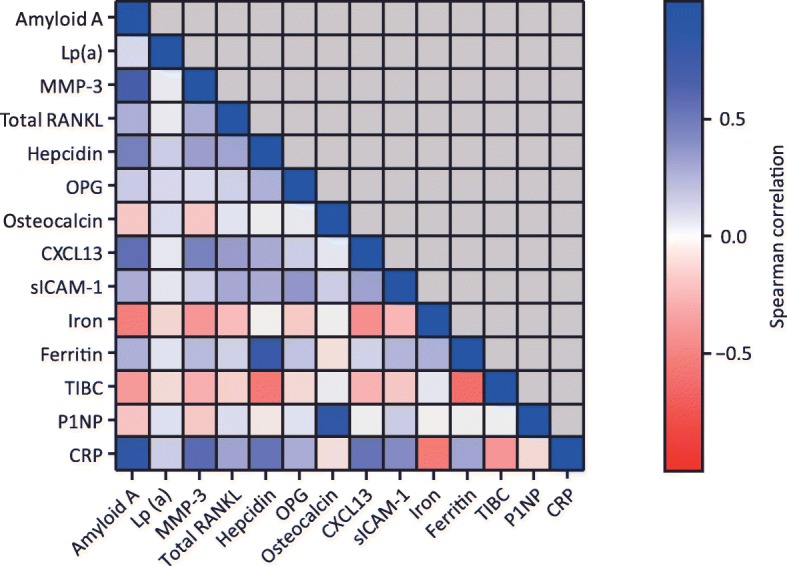
Correlations between baseline biomarkers. CRP, C-reactive protein; CXCL13, chemokine (C-X-C motif) ligand 13; Lp(a), lipoprotein (a); MMP-3, matrix metalloproteinase-3; OPG, osteoprotegerin; P1NP, procollagen type 1 *N*-terminal propeptide; RANKL, receptor activator of nuclear factor-κB ligand; SAA, serum amyloid A; sICAM-1, soluble intercellular adhesion molecule-1; TIBC, total iron-binding capacity

Correlations between individual biomarkers at baseline were generally low or moderate (*ρ* < 0.5; Fig. 1). Correlation coefficients above 0.7 were observed for markers of inflammation (CRP and SAA; *ρ* = 0.81), bone formation (P1NP and OC; *ρ* = 0.82) and anaemia of chronic disease (ferritin and hepcidin; *ρ* = 0.74), as expected. Moderate correlations were observed between baseline CRP, SAA or MMP-3 with differential blood counts (leucocytes and neutrophils; *ρ* from 0.4 to 0.5) and, as expected, between iron and haemoglobin (*ρ* = 0.57; Figure S1).

### Pharmacodynamic effects of treatment on biomarkers

**Fig. 2:**
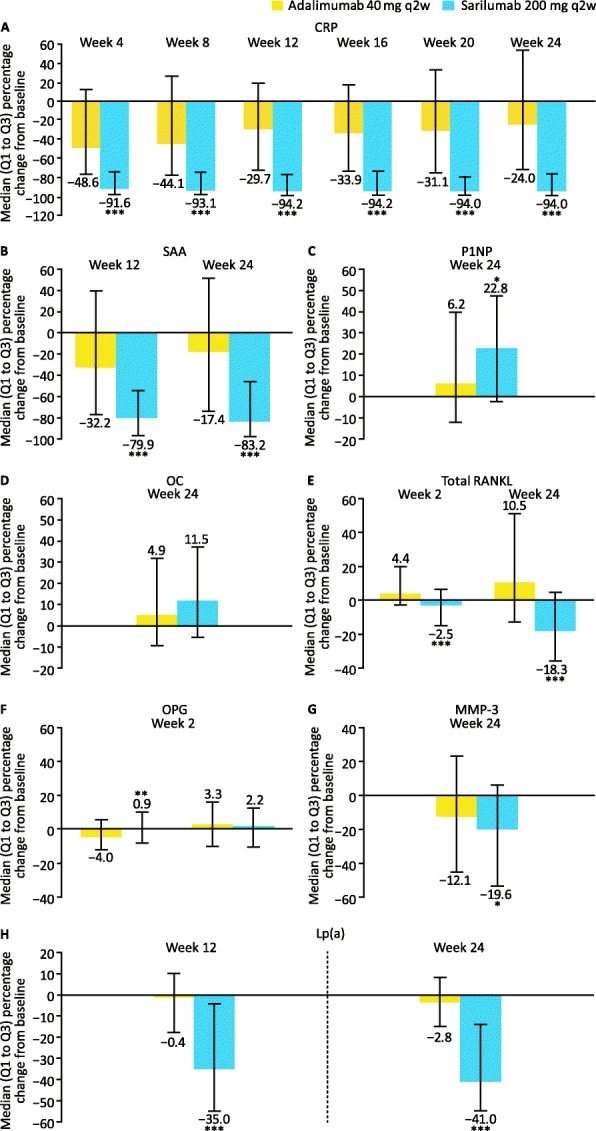
Median percentage changes from baseline in biomarkers through week 24. Median percentage changes from baseline in biomarkers of **a**, **b** the acute-phase response (CRP and SAA), **c**–**f** bone remodelling (P1NP, OC, total RANKL and OPG), **g** synovial inflammation (MMP-3) and **h** atherothrombosis (Lp [a]). *Adjusted *p* < 0.05; **adjusted *p* < 0.01 vs. adalimumab; ***adjusted *p* < 0.0001 vs. adalimumab (Benjamini–Hochberg procedure). CRP, C-reactive protein; Lp(a), lipoprotein (a); MMP-3, matrix metalloproteinase-3; OC, osteocalcin; OPG, osteoprotegerin; P1NP, procollagen type 1 *N*-terminal propeptide; Q, quartile; q2w, every 2 weeks; RANKL, receptor activator of nuclear factor-κB ligand; SAA, serum amyloid A

To compare the effects of sarilumab and adalimumab treatment on biomarkers over time, the absolute (Table S4) and percentage changes from baseline in biomarker concentrations were analysed up to week 24. Greater reductions in biomarkers associated with the acute-phase response were observed at weeks 12 and 24 following treatment with sarilumab vs. adalimumab (adjusted *p* < 0.0001; Fig. 2a, b). Reductions in CRP were observed as early as week 4 with sarilumab vs. adalimumab and were sustained throughout the treatment period (Fig. 2a for median percentage change in CRP from baseline in biomarker population from week 4; Table S5 for percentage of patients with CRP ≤ 10 mg/L and ≤ 3 mg/L at weeks 12 and 24 [observed cases within the ITT population]).

At week 24, sarilumab treatment increased concentrations of P1NP, a marker of osteoblast activation, compared with adalimumab (adjusted *p* = 0.027; Fig. 2c). A numeric increase in OC, another marker of osteoblast activity, was also observed in sarilumab- vs. adalimumab-treated patients (Fig. 2d). Furthermore, reductions in total RANKL, a marker of bone remodelling, were observed as early as week 2 with sarilumab compared with adalimumab and persisted through week 24 (adjusted *p* < 0.0001; Fig. 2e); in addition, a numeric increase in total RANKL was observed after adalimumab treatment. A transient decrease in OPG, a decoy for RANKL, was observed after adalimumab treatment at week 2 but did not persist through week 24 (Fig. 2f). The log of the ratio of RANKL to OPG was also significantly decreased in patients treated with sarilumab vs. adalimumab (data not shown). Subgroup analyses revealed that this effect was significant through week 24 in patients who were not on steroids at baseline (nominal *p* = 0.0013). Additionally, greater reductions in MMP-3 were observed with sarilumab at week 24 (adjusted *p* = 0.020; Fig. 2g). Since corticosteroid use impacts bone remodelling, we compared the treatment effects in subgroups based on baseline corticosteroid use. At week 24 the differential effects of sarilumab on MMP-3 concentrations were significant (nominal *p* = 0.001) only in the subgroup of patients (sarilumab: *n* = 79, 51.6%; adalimumab: *n* = 88, 57.1%) who were not on concomitant steroids (data not shown). Reductions in total RANKL were significant in the sarilumab treatment group compared with adalimumab treatment irrespective of steroid use at baseline (nominal *p* < 0.01). Increases in P1NP, though numerically increased by sarilumab treatment relative to adalimumab, did not reach statistical significance in the steroid subgroups (median percentage change from baseline in P1NP [interquartile range]: with baseline steroid use, 24.6% [− 0.6 to 50.3%] with sarilumab and 7.5% [− 9.9 to 40.3%] with adalimumab [nominal *p* = 0.0757]; without baseline steroid use, 18.0% [− 3.5 to 47.2%] with sarilumab and 1.8% [− 13.9 to 31.9%] with adalimumab [nominal *p* = 0.1625]).

The effects of sarilumab and adalimumab on biomarkers associated with markers purported to reflect synovial lymphoid and myeloid cell infiltrates, CXCL13 and sICAM-1, respectively, were also examined. While greater reductions in these biomarkers were observed 2 weeks post-treatment with adalimumab vs. sarilumab, these effects did not persist through week 24 (Figure S2).

We also examined the effects of treatment on parameters associated with anaemia of chronic disease. Previously unpublished data showed that, in the overall safety population (sarilumab, *n* = 184; adalimumab, *n* = 185), sarilumab resulted in larger increases in haemoglobin levels vs. adalimumab [20] from baseline (mean 13.0 and 12.9 g/dL, respectively) at week 12 (LSM changes from baseline 0.53 vs. 0.12 g/dL, respectively; LSM difference 0.41 g/dL [95% CI 0.22–0.60; nominal *p* < 0.001]) and week 24 (LSM changes from baseline 0.59 vs. 0.08 g/dL, respectively; LSM difference 0.52 g/dL [95% CI 0.32–0.71; nominal *p* < 0.001]). Furthermore, in the overall ITT population, a numerically greater reduction from baseline in the proportion of patients with anaemia was observed with sarilumab vs. adalimumab from baseline: reductions were observed as early as week 2 and persisted through week 24 (Table S6). In this post hoc analysis, reductions in hepcidin and ferritin were observed at week 2 with both sarilumab and adalimumab. In contrast, increases in iron and TIBC were observed with sarilumab relative to adalimumab at week 2 post-treatment (Figure S3). Reductions in the lipid particle Lp(a) were observed with sarilumab vs. adalimumab at week 24 (adjusted *p* < 0.0001; Fig. 2h).

**Fig. 3:**
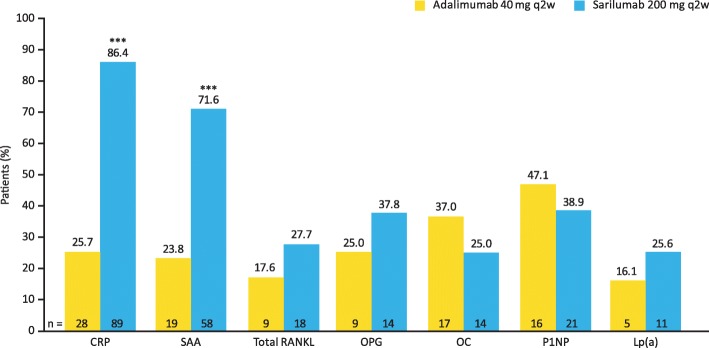
Proportions of patients whose biomarker values returned to normal reference ranges at week 24. Proportions of patients with biomarker serum concentrations exceeding the reference range at baseline that normalized to within reference range at week 24. ***Nominal *p* < 0.0001 vs. adalimumab (*X*^2^ test). CRP, C-reactive protein; Lp(a), lipoprotein (a); OC, osteocalcin; OPG, osteoprotegerin; P1NP, procollagen type 1 *N*-terminal propeptide; q2w, every 2 weeks; RANKL, receptor activator of nuclear factor-κB ligand; SAA, serum amyloid A

A subset of patients had abnormal baseline biomarker levels relative to reference ranges. In these patients, normalization of CRP and SAA was evident in a greater percentage treated with sarilumab than adalimumab at week 24 (nominal *p* < 0.0001). Normalization of total RANKL, OPG and Lp(a) occurred in a numerically greater percentage of patients treated with sarilumab vs. adalimumab at week 24 (Fig. 3).

### Relationship between changes in biomarker levels and clinical responses

To establish whether post-treatment changes in biomarker levels at week 24 were associated with clinical efficacy, changes were compared between sarilumab- and adalimumab-treated responders and non-responders. Median percentage changes at week 24 in total RANKL, OPG, P1NP, OC and Lp(a) did not differ greatly between responders and non-responders (data not shown). However, reductions in SAA from baseline at week 24 were greater in adalimumab ACR20 and DAS28-CRP < 3.2 responders than non-responders (− 33.3% vs. 0.0%, respectively; nominal *p* = 0.0038 and − 39.2% vs. 0.0%, respectively; nominal *p* = 0.0061, respectively). Greater reductions in MMP-3 were also observed in adalimumab ACR20 responders vs. non-responders (− 23.6% vs. 17.1%, respectively; nominal *p* < 0.0001). Associations between clinical efficacy and changes from baseline in SAA and MMP-3 were not observed in sarilumab-treated patients, and although both responders and non-responders had a ≥ 90% reduction in CRP, the *p* values for comparisons of responders vs. non-responders were > 0.05 across several parameters, including ACR20/50, DAS28-CRP < 3.2 and DAS28-CRP < 2.6 (data not shown).

### Correlations between biomarkers and disease activity and PROs at baseline

The strongest correlations between baseline biomarkers and baseline disease activity were observed for SAA and CRP with DAS28-ESR (*ρ* = 0.26 and 0.31, respectively) and for CRP, SAA, MMP-3, hepcidin and CXCL13 with DAS28-CRP (*ρ* from 0.36 to 0.58). None of the biomarkers correlated with baseline PROs (all *ρ* < 0.3).

### Predictive analysis of baseline biomarker levels on clinical responses and PROs

**Fig. 4:**
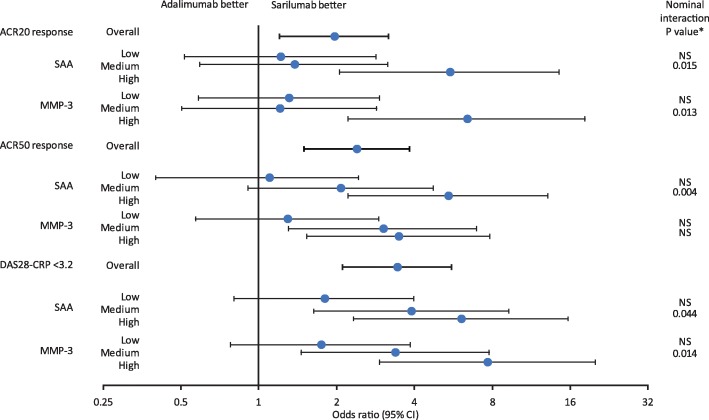
Odds ratios for achieving efficacy endpoints at week 24 by baseline biomarker tertile. Odds ratios (sarilumab vs. adalimumab) for achieving ACR20, ACR50 and DAS28-CRP < 3.2 responses at week 24 by baseline biomarker tertile. *Nominal biomarker-by-treatment interaction vs. low tertile. Low, medium and high subgroups are based on biomarker tertile values in overall treatment groups (see Table S3 for tertile ranges). ACR20/50, American College of Rheumatology 20/50% improvement criteria; CI, confidence interval; DAS28-CRP, Disease Activity Score (28 joints) using C-reactive protein; MMP-3, matrix metalloproteinase-3; NS, not significant at 5%; SAA, serum amyloid A

Baseline biomarker levels were analysed as continuous and categorical measures by tertiles (low, medium and high) because thresholds associated with clinical efficacy are not currently established, and treatment-by-biomarker interaction *p* values were calculated to assess the predictivity of the biomarker. Treatment-by-tertile biomarker interactions for efficacy endpoints at week 24 analysed by baseline biomarker in tertiles are shown in Fig. 4 and Table S7. Patients with the highest baseline concentrations of SAA who received sarilumab were more likely to achieve ACR20/50/70 or DAS28-CRP < 3.2 responses than with adalimumab compared with patients in the low tertile: ACR20 (OR [95% CI] 5.5 [2.1, 14.5]), ACR50 (5.4 [2.2, 13.2]), ACR70 (5.7 [1.8, 18.4]) and DAS28-CRP < 3.2 (6.1 [2.3, 15.7]) (Fig. 4 and Table S7). SAA was consistently predictive compared with high MMP-3 and CRP, which were only predictive of ACR20 and DAS28-CRP < 3.2 response (Table S7). Baseline levels of biomarkers associated with bone remodelling, synovial lymphoid and myeloid cell infiltrates and anaemia of inflammation were not predictive of efficacy at week 24, except for hepcidin and CXCL13, which were associated with ACR20 response.

**Fig. 5:**
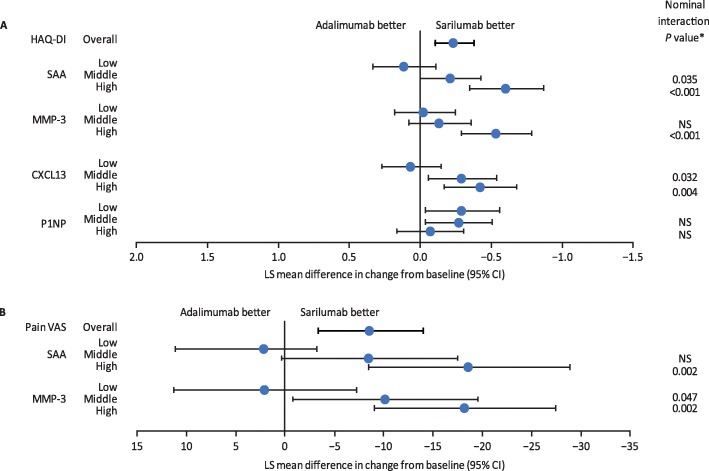
Changes from baseline in PROs at week 24 by baseline biomarker tertile. LS mean differences in changes from baseline between sarilumab and adalimumab at week 24 by baseline biomarker tertile in **a** HAQ-DI and **b** pain VAS. *Nominal biomarker-by-treatment interaction vs. low tertile. Low, medium and high subgroups are based on biomarker tertile values in overall treatment groups (see Table S3 for tertile ranges). CI, confidence interval; CXCL13, chemokine (C-X-C motif) ligand 13; HAQ-DI, Health Assessment Questionnaire-Disability Index; LS, least squares; MMP-3, matrix metalloproteinase-3; NS, not significant at 5%; P1NP, procollagen type 1 *N*-terminal propeptide; SAA, serum amyloid A; VAS, visual analogue scale

The ability of baseline biomarker levels to predict PRO responses was also analysed by their respective tertiles and showed that sarilumab-treated patients with higher SAA, MMP-3 and hepcidin levels reported improved PRO responses including HAQ-DI (Fig. 5a) and pain VAS (Fig. 5b) scores compared with adalimumab-treated patients, as well as patient global VAS, morning stiffness VAS, SF-36 PCS and physical functioning domain and RAID. The *p* values for these interactions are included in Fig. 5 and Table S8 to demonstrate the differential efficacy predicted by high levels of these biomarkers compared with low levels. Baseline levels of markers associated with anaemia of chronic disease (hepcidin, ferritin and iron) were also associated with PRO improvements at week 24 (Table S8). Analysis of biomarkers as continuous measures also revealed interactions for SAA, MMP-3, CRP and P1NP with HAQ-DI at week 24 (interaction nominal *p* values < 0.01).

### Evaluation of differential combinations of markers associated with myeloid and lymphoid activation for prediction of clinical response

Baseline levels of CXCL13 and sICAM-1 were analysed to determine whether differential ratios of these biomarkers (high/high, high/low, low/high and low/low; using the median in the overall population as the cut-off) could predict clinical responses to sarilumab or adalimumab treatment at week 24. While CXCL13 high/sICAM-1 high and CXCL13 low/sICAM-1 low patients had greater ACR50 responses with sarilumab than adalimumab, the other combinations were not predictive (nominal *p* > 0.05; Figure S4). Additionally, CXCL13 high/sICAM-1 high patients had greater ACR20 responses with sarilumab than adalimumab (OR 3.8 [95% CI 1.5, 9.8]; nominal *p* = 0.004) but other combinations were not predictive (nominal *p* > 0.05).

## Discussion

Analysis of the pharmacodynamic effects of treatment on circulating biomarkers found sarilumab treatment reduced biomarkers of the acute-phase response, bone remodelling, synovial inflammation and CV risk and increased bone formation markers compared with adalimumab. These effects were generally observed early and persisted through to week 24. This was particularly evident with CRP and is consistent with previous observations [13, 14, 18, 19]. In addition, a greater proportion of patients treated with sarilumab vs. adalimumab monotherapy demonstrated normalization of serum biomarkers at week 24, which was greatest for biomarkers of the acute-phase response (CRP and SAA).

In this study, a limited number of bone remodelling markers were evaluated. Inhibitors of bone formation induced by the Wnt pathway such as DKK-1 and sclerostin are upregulated in patients with RA and have been shown to be decreased by anti-IL-6 and anti-TNF therapies [21]. The pharmacodynamic effects of sarilumab monotherapy observed in MONARCH are generally consistent with those reported after 24 weeks treatment in the MOBILITY (MTX-IR patients treated with sarilumab 150 mg/200 mg q2w + MTX vs. placebo + MTX) and TARGET (TNF-IR patients treated with sarilumab 150 mg/200 mg q2w + csDMARDs vs. placebo + csDMARDs) biomarker populations, where sarilumab treatment reduced biomarkers of bone resorption and synovial inflammation [18, 19]. P1NP was significantly increased after sarilumab monotherapy compared with adalimumab, but in other studies, TNF inhibition has been associated with significant increases in P1NP post-treatment [22].

Limited data in patients with RA suggest that the IL-6 receptor blocker tocilizumab was more effective than TNF-α inhibitors in improving anaemia of chronic disease and normalizing iron metabolism via inhibition of hepcidin [23]. As hepcidin is involved in regulating iron stores in macrophages, a reduction in hepcidin is associated with greater iron availability. We observed increases in both iron and TIBC levels with sarilumab vs. adalimumab treatment despite similar effects of both on hepcidin and ferritin.

In our analysis, given that both SAA and CRP are regulated by IL-6, biomarkers of the acute-phase response were strongly correlated with DAS28-CRP at baseline (nominal *p* < 0.0001). However, no correlations were observed between baseline biomarkers and PROs. Reductions in several biomarkers were associated with clinical efficacy at week 24 in adalimumab-treated patients; however, these associations were not observed in the sarilumab group. This result suggests that IL-6 receptor blockade may have a direct effect on production of these biomarkers independent of its effects on disease activity, in contrast to TNF inhibitors.

Biomarkers that can predict responses to treatment would be valuable in supporting the paradigm shift towards personalized medicine [24–26]. In this study, univariate analysis identified several biomarkers that individually predicted responses to sarilumab treatment. Upon further analysis, high levels of CRP, SAA, MMP-3, CXCL13 and hepcidin predicted ACR20 responses to sarilumab. Several of these markers are included in the multi-biomarker disease activity (MBDA) score which is associated with disease activity but has not been evaluated for differential response to specific biologic therapies [27, 28]. Baseline levels of markers associated with anaemia of chronic disease did not predict efficacy (except hepcidin); however, these markers were associated with changes in several PROs. High baseline levels of SAA and MMP-3 were also associated with improvements in several PROs, including patient global VAS, HAQ-DI, pain VAS, SF-36 PCS and MCS scores, morning stiffness VAS and RAID score.

Dennis et al. observed that patients with greater ACR50 response rates following treatment with the IL-6 inhibitor tocilizumab had higher baseline lymphoid relative to myeloid activity [27]. Conversely, patients with higher myeloid synovial signatures responded better to adalimumab, suggesting that baseline characteristics may predict therapeutic responses to agents with different mechanisms of action. We also evaluated whether differential levels of CXCL13 and sICAM-1 predicted ACR50 responses in MONARCH. We used similar methodology to Dennis et al. (defining low and high levels using the pre-treatment median as the cut-off) but owing to different biomarker levels in patient populations, medians were not comparable, and our results did not replicate the previous findings. This may also be attributed to baseline differences in synovial phenotypes between the two trial populations and/or differences in the assays used to measure these markers. Synovial biopsies were not performed before initiating therapy in either trial, and although synovial phenotypes can predict differential responses, other markers reflective of lymphoid and myeloid cells may require measurement to capture underlying synovial phenotypes and pathology.

Interpretations of this analysis should acknowledge these data are up to week 24 following treatment initiation and the limited sampling of biomarkers (many only at baseline and weeks 2 and 24); further exploration of more frequent and/or later timepoints may provide a more complete picture of biomarker changes following therapy. It is also important to recognize that these analyses were restricted to circulating biomarkers accounting for only a portion of total biomarker matter; additional approaches are needed to understand the effects of treatment on synovial biomarkers. Finally, this was a post hoc analysis of patients well defined according to inclusion and exclusion criteria of the trial; therefore, additional analyses are required to further understand the effect of sarilumab treatment on these biomarkers in a wider population of RA patients.

## Conclusion

In this analysis, sarilumab treatment was associated with decreases in circulating biomarkers of the acute-phase response, bone resorption, synovial inflammation and CV risk compared with adalimumab. Several biomarkers, including MMP-3, SAA and CRP, were associated with clinical efficacy and individually predicted response to sarilumab treatment. Further studies evaluating the predictive value of changes in these biomarkers are necessary to confirm these findings and identify patients more likely to respond to sarilumab administration.

## Supplementary information

**Additional file 1. **Additional methodology. **Table S1.** Individual serum biomarker assessment schedule. **Table S2.** Efficacy and PROs at week 24 in the biomarker and ITT populations. **Table S3.** Baseline biomarker serum concentrations in the biomarker population. **Table S4.** Absolute change from baseline in biomarker concentrations through week 24. **Table S5.** Percentage of patients with CRP ≤10 mg/L and ≤3 mg/L at weeks 12 and 24 (overall safety population). **Table S6.** Percentage of patients with anaemia at weeks 2 and 24 (overall safety population). **Table S7.** Treatment-by-tertile biomarker interactions for efficacy endpoints at week 24 analysed by baseline biomarker in tertiles. **Table S8.** Treatment-by-tertile biomarker interactions for PROs at week 24 analysed by baseline biomarker in tertiles. **Figure S1.** Correlation matrix for baseline biomarkers and haematology parameters. **Figure S2.** Median percentage changes from baseline in (A) CXCL13 and (B) sICAM-1 through week 24. **Figure S3.** Median percentage changes from baseline in biomarkers of anaemia of chronic disease 2 weeks post treatment. **Figure S4.** ACR50 responses at week 24 and corresponding ORs with differential combinations of CXCL13 and sICAM-1.

## Data Availability

Qualified researchers may request access to patient-level data and related study documents, including the clinical study report, study protocol with any amendments, blank case report form, statistical analysis plan and dataset specifications. Patient-level data will be anonymized and study documents will be redacted to protect the privacy of trial participants. Further details on Sanofi’s data-sharing criteria, eligible studies and process for requesting access can be found at https://www.clinicalstudydatarequest.com.

## Abbreviations

ACPA: Anti-citrullinated protein antibody
ACR20/50/70: American College of Rheumatology 20/50/70% improvement criteria
ANCOVA: Analysis of covariance
ANOVA: Analysis of variance
CDAI: Clinical Disease Activity Index
CI: Confidence interval
CRP: C-reactive protein
csDMARD: Conventional synthetic disease-modifying anti-rheumatic drug
CXCL13: Chemokine (C-X-C motif) ligand 13
DAS28-CRP: Disease Activity Score (28 joints) using C-reactive protein
CV: Cardiovascular
DAS28-ESR: Disease Activity Score (28 joints) using erythrocyte sedimentation rate
FACIT: Functional Assessment of Chronic Illness Therapy
HAQ-DI: Health Assessment Questionnaire-Disability Index
IL-6: Interleukin-6
ITT: Intent-to-treat
Lp(a): Lipoprotein (a)
LS: Least squares
LSM: Least squares mean
MBDA: Multi-biomarker disease activity
MCS: Mental component summary
MMP-3: Matrix metalloproteinase-3
MTX-INT/MTX-IR: Intolerant of or inadequate responders to methotrexate
OC: Osteocalcin
OPG: Osteoprotegerin
OR: Odds ratio
P1NP: Procollagen type 1 *N*-terminal propeptide
PCS: Physical component summary
PRO: Patient-reported outcome
q2w: Every 2 weeks
RA: Rheumatoid arthritis
RAID: Rheumatoid arthritis impact of disease
RANKL: Receptor activator of nuclear factor-κB ligand
RF: Rheumatoid factor
SAA: Serum amyloid A
SF-36: Medical Outcomes Study Short-Form (36-item) Health Survey
sICAM-1: Soluble intercellular adhesion molecule-1
SJC: Swollen joint count
TIBC: Total iron-binding capacity
TJC: Tender joint count
TNF-α: Tumour necrosis factor alpha
VAS: Visual analogue scale
