# Supplementary material for: Sarilumab and adalimumab differential effects on bone remodelling and cardiovascular risk biomarkers, and predictions of treatment outcomes
Source: Arthritis Res Ther. 2020 Apr 7;22:70. doi: 10.1186/s13075-020-02163-6 (PMC7137491; doi:10.1186/s13075-020-02163-6)
Supplement: Supplementary file 1 — Additional file 1. Additional methodology. Table S1. Individual serum biomarker assessment schedule. Table S2. Efficacy and PROs at week 24 in the biomarker and ITT populations. Table S3. Baseline biomarker serum concentrations in the biomarker population. Table S4. Absolute change from baseline in biomarker concentrations through week 24. Table S5. Percentage of patients with CRP ≤10 mg/L and ≤3 mg/L at weeks 12 and 24 (overall safety population). Table S6. Percentage of patients with anaemia at weeks 2 and 24 (overall safety population). Table S7. Treatment-by-tertile biomarker interactions for efficacy endpoints at week 24 analysed by baseline biomarker in tertiles. Table S8. Treatment-by-tertile biomarker interactions for PROs at week 24 analysed by baseline biomarker in tertiles. Figure S1. Correlation matrix for baseline biomarkers and haematology parameters. Figure S2. Median percentage changes from baseline in (A) CXCL13 and (B) sICAM-1 through week 24. Figure S3. Median percentage changes from baseline in biomarkers of anaemia of chronic disease 2 weeks post treatment. Figure S4. ACR50 responses at week 24 and corresponding ORs with differential combinations of CXCL13 and sICAM-1. [file 13075_2020_2163_MOESM1_ESM.docx]

## Supplementary data

Additional methodology

### Biomarker analysis

All biomarker serum concentrations, except C-reactive protein (CRP), were analysed retrospectively using a validated proprietary enzyme-linked immunosorbent assay (ELISA) at Bioclinica Lab (Lyon, France). CRP was assessed at Covance Laboratories (Indianapolis, IN, USA, Geneva, Switzerland or Singapore) using the Siemens high-sensitivity CRP nephelometry assay. The intra-assay precision was <3%, inter-assay precision was <5.4%, and the reference range for healthy controls was ≤2.87 mg/L. Serum levels of chemokine (C-X-C motif) ligand 13 and soluble intercellular adhesion molecule-1 were assessed using an ELISA (Quantikine^®^ ELISA kit, R&D Systems, Minneapolis, MN, USA), with inter-assay coefficient of variation (CV) <8% and intra-assay CV <15%. Serum procollagen type 1 *N*-terminal propeptide (P1NP) was measured using the Roche Modular S P1NP assay, with intra- and inter-assay CVs <7%. Serum amyloid A was measured using an ELISA (Anogen) with intra- and inter-assay CVs <7%. Ferritin was measured using the Roche Modular Serum Ferritin assay, with intra- and inter-assay CVs <3%. Total iron-binding capacity was measured using a Kone 20 analyser, Konelab (Total Iron-Binding Capacity [RANDOX]) with intra- and inter-assay CVs <5.5% and <4.7%, respectively. Serum levels of iron were measured using a Kone 20 analyser, Konelab (Iron [Thermo Scientific]) with intra- and inter-assay CVs <7.8% and <6%, respectively. Hepcidin levels were measured using an ELISA (Human Hepcidin 25 (bioactive) HS ELISA [DRG]) with intra- and inter-assay CVs <9.6% and <8.1%, respectively. Biomarker levels below the lower limit of quantification (LLOQ) were replaced by LLOQ/2 in all analyses, and those above upper limit of quantification (ULOQ) by ULOQ.

## Supplementary tables and figures

**Table S1** Individual serum biomarker assessment schedule

**Table S2** Efficacy and PROs at week 24 in the biomarker and ITT populations

**Table S3** Baseline biomarker serum concentrations in the biomarker population

**Table S4** Absolute change from baseline in biomarker concentrations through week 24

**Table S5** Percentage of patients with CRP ≤10 mg/L and ≤3 mg/L at weeks 12 and 24 (ITT population)

**Table S6** Percentage of patients with anaemia at weeks 2 and 24 (ITT population)

**Table S7** Treatment-by-tertile biomarker interactions for efficacy endpoints at week 24 analysed by baseline biomarker in tertiles

**Table S8** Treatment-by-tertile biomarker interactions for PROs at week 24 analysed by baseline biomarker in tertiles

**Fig. S1** Correlation matrix for baseline biomarkers and haematology parameters

**Fig. S2** Median percentage changes from baseline in (A) CXCL13 and (B) sICAM-1 through week 24

**Fig. S3** Median percentage changes from baseline in biomarkers of anaemia of chronic disease 2 weeks post treatment

**Fig. S4** ACR50 responses at week 24 and corresponding ORs with differential combinations of CXCL13 and sICAM-1

**Table S1** Individual serum biomarker assessment schedule

| **Function** | **Biomarker** | **Baseline** | **Week 2** | **Week 4** | **Week 8** | **Week 12** | **Week 16** | **Week 20** | **Week 24** |
| --- | --- | --- | --- | --- | --- | --- | --- | --- | --- |
| Acute-phase response | SAA | X |  |  |  | X |  |  | X |
|  | CRP | X |  | X | X | X | X | X | X |
| Atherothrombosis | Lp(a) | X |  |  |  | X |  |  | X |
| Synovial inflammation | MMP-3 | X |  |  |  |  |  |  | X |
| Bone remodelling | Total RANKL | X | X |  |  |  |  |  | X |
|  | P1NP | X |  |  |  |  |  |  | X |
|  | OPG | X | X |  |  |  |  |  | X |
|  | Osteocalcin | X |  |  |  |  |  |  | X |
| Marker reflecting synovial lymphoid cell infiltrate  Marker reflecting synovial myeloid cell infiltrate | CXCL13 | X | X |  |  |  |  |  | X |
|  | sICAM-1 | X | X |  |  |  |  |  | X |
| Anaemia of chronic disease | Iron | X | X |  |  |  |  |  |  |
|  | Ferritin | X | X |  |  |  |  |  |  |
|  | TIBC | X | X |  |  |  |  |  |  |
|  | Hepcidin | X | X |  |  |  |  |  |  |

CRP: C-reactive protein; CXCL13: chemokine (C-X-C motif) ligand 13; Lp(a): lipoprotein (a); MMP-3: matrix metalloproteinase-3; OPG: osteoprotegerin; P1NP: procollagen type 1 *N*-terminal propeptide; RANKL: receptor activator of nuclear factor-κB ligand; SAA: serum amyloid A; sICAM-1: soluble intercellular adhesion molecule-1; TIBC: total iron-binding capacity.

| **Table S2** Efficacy and PROs at week 24 in the biomarker and ITT populations | | | | |
| --- | --- | --- | --- | --- |
|  | **ITT population** | | **Biomarker population** | |
|  | **Adalimumab  40 mg q2w (*n* = 185)** | **Sarilumab  200 mg q2w (*n* = 184)** | **Adalimumab  40 mg q2w (*n* = 154)** | **Sarilumab  200 mg q2w (*n* = 153)** |
| **Efficacy results at week 24^a^** | | | | |
| ACR20 responders, % | 58.4 | 71.7 | 59.1 | 73.9 |
| ACR50 responders, % | 29.7 | 45.7 | 29.2 | 49.7 |
| ACR70 responders, % | 11.9 | 23.4 | 12.3 | 25.5 |
| ΔDAS28-ESR | –2.2 (1.4) | –3.4 (1.4) | –2.3 (1.3) | –3.4 (1.3) |
| DAS28-ESR <2.6, % | 7.0 | 26.6 | 8.4 | 27.5 |
| DAS28-ESR <3.2, % | 14.1 | 42.9 | 15.6 | 43.8 |
| ΔDAS28-CRP | –2.1 (1.2) | –2.9 (1.3) | –2.1 (1.3) | –3.0 (1.2) |
| DAS28-CRP <2.6, % | 13.5 | 34.2 | 13.0 | 34.6 |
| DAS28-CRP <3.2, % | 24.3 | 51.6 | 24.0 | 52.3 |
| ΔTJC | –16.4 (12.0) | –19.0 (13.3) | –16.6 (12.3) | –19.4 (12.5) |
| ΔSJC | –12.2 (9.1) | –14.3 (9.6) | –12.1 (8.9) | –14.6 (9.8) |
| ΔCDAI | –25.5 (12.9) | –29.7 (12.7) | –25.9 (13.3) | –30.2 (12.0) |
| CDAI ≤2.8, % | 2.7 | 7.1 | 3.2 | 7.2 |
| CDAI ≤10, % | 24.9 | 41.8 | 24.7 | 43.1 |
| ΔPhysician global  VAS (0–100 mm) | –37.3 (22.5) | –45.3 (21.4) | –37.6 (22.8) | –45.7 (20.5) |
| **PROs at week 24^a^** | | | | |
| ΔHAQ-DI | –0.4 (0.6) | –0.6 (0.7) | –0.4 (0.6) | –0.7 (0.7) |
| ΔFACIT-Fatigue  (0–52) | 8.2 (10.4) | 10.4 (10.2) | 8.4 (10.3) | 11.3 (10.0) |
| ΔPatient global  VAS (0–100 mm) | –25.0 (25.2) | –33.5 (26.2) | –25.5 (24.9) | –33.8 (26.3) |
| ΔPain VAS  (0–100 mm) | –27.9 (24.7) | –36.4 (26.9) | –28.0 (24.4) | –36.9 (27.0) |
| ΔSF-36 PCS | 5.5 (7.1) | 8.6 (7.7) | 5.3 (7.1) | 9.0 (7.6) |
| ΔSF-36 MCS | 7.0 (11.3) | 8.2 (10.8) | 6.9 (11.0) | 8.5 (11.3) |
| ΔMorning stiffness VAS  (0–100 mm) | –27.0 (27.4) | –36.1 (27.9) | –26.7 (27.8) | –36.7 (27.3) |
| ΔRAID (0–10) | –2.1 (2.4) | –3.2 (2.4) | –2.1 (2.4) | –3.3 (2.4) |

^a^Mean (standard deviation) unless otherwise stated. Δ: absolute change from baseline; ACR20/50/70: American College of Rheumatology 20/50/70% improvement criteria; CDAI: Clinical Disease Activity Index; DAS28-CRP: Disease Activity Score (28 joints) using C-reactive protein; DAS28-ESR: Disease Activity Score (28 joints) using erythrocyte sedimentation rate; FACIT: Functional Assessment of Chronic Illness Therapy; HAQ-DI: Health Assessment Questionnaire-Disability Index; ITT: intent-to-treat; MCS: mental component summary; PCS: physical component summary; PRO: patient-reported outcome; q2w: every 2 weeks; RAID: rheumatoid arthritis impact of disease; SJC: swollen joint count; TJC: tender joint count; VAS: visual analogue scale.

**Table S3** Baseline biomarker serum concentrations in the biomarker population

| **Biomarker** | **Adalimumab  40 mg q2w  (*n* = 154)** | **Sarilumab 200 mg q2w (*n* = 153)** | **Low tertile** | **Medium tertile** | **High tertile** | **Reference range^a^** |
| --- | --- | --- | --- | --- | --- | --- |
| SAA, ng/L | 22 806.0  (5817.2, 115 200.0) | 16 089.0  (4997.5, 85 918.0) | 3734.7  (2192.7, 5346.4) | 18549.5  (11 832.0, 30 082.0) | 174 900.0 (105 200.0, 256 000.0) | 1000.0–9249.3 |
| CRP, mg/L | 9.4 (3.8, 33.5) | 7.8 (2.8, 24.7) | 1.9 (1.0, 3.4) | 8.5 (6.9, 13.1) | 37.6 (27.9, 65.1) | ≤2.9 |
| Lp(a), mg/L | 235.5 (111.0, 559.0) | 179.0 (78.0, 402.0)* | 48.5  (17.5, 100.0) | 192.0  (157.0, 236.0) | 689.5  (450.0, 1116.0) | 19.0–1028.0 |
| MMP-3, ng/mL | 44.0 (25.2, 80.9) | 40.8 (19.3, 74.4) | 16.0 (10.3, 20.8) | 42.8 (35.5, 54.1) | 99.9 (77.0, 154.3) | 6.0–15.8 |
| Total RANKL, pmol/L | 484.5 (254.7, 1423.1) | 547.6 (268.5, 1361.3) | 200.1  (136.7, 258.5) | 515.0  (424.3, 674.0) | 2252.8  (1417.6, 3657.6) | 35.1–639.7 |
| P1NP, ng/mL | 45.9 (34.9, 63.9) | 44.7 (30.3, 59.9) | 27.6 (21.4, 32.9) | 45.6 (41.6, 50.1) | 73.2 (63.0, 87.6) | 47.9–204.1 |
| OPG, pmol/L | 5.9 (5.0, 8.0) | 6.0 (4.7, 7.5) | 4.3 (3.9, 5.0) | 5.9 (5.6, 6.5) | 8.8 (7.7, 10.5) | 3.6–7.9 |
| OC, ng/mL | 19.0 (13.8, 26.0) | 18.0 (13.9, 25.8) | 12.0 (9.6, 13.8) | 18.6 (16.8, 21.1) | 28.9 (26.0, 35.6) | 13.9–30.6 |
| CXCL13, pg/mL | 120.1 (72.4, 184.7) | 112.8 (70.8, 180.8) | 61.8 (52.4, 72.0) | 116.4 (98.2, 130.6) | 236.8 (180.8, 323.9) | 37.8–153.6 |
| sICAM-1, ng/mL | 258.6 (212.1, 324.8) | 257.3 (212.7, 304.0) | 199.3 (179.7, 212.1) | 257.7 (239.7, 272.3) | 339.4 (313.7, 380.0) | 186.0–331.0 |
| Iron μmol/L | 10.5 (7.0, 14.9) | 11.3 (7.2, 16.0) | 6.1 (4.2, 7.0) | 10.9 (9.8, 12.2) | 17.2 (15.5, 20.3) | 10.8–28.9 |
| Ferritin, ng/mL | 80.0 (41.1, 174.0) | 74.9 (35.1, 130.6) | 24.9 (13.9, 35.5) | 76.7 (60.5, 93.5) | 204.3 (154.9, 283.4) | 18.6–148.3 |
| TIBC, μg/dL | 321.5 (293.5, 350.5) | 324.0 (303.0, 361.0) | 286.0 (267.0, 297.0) | 322.0 (313.0, 332.0) | 373.0 (357.0, 397.0) | 247.2–363.0 |
| Hepcidin, ng/mL | 24.8 (9.7, 48.9) | 20.9 (9.2, 39.3) | 6.0 (3.7, 9.3) | 23.0 (17.0, 28.9) | 62.4 (43.9, 77.0) | 0.6–46.4 |

Data presented as median (Q1, Q3) at baseline. ^a^Reference range for CRP is based on population of healthy men and women (reference range provided by Covance); for all other biomarkers, reference range is based on healthy post-menopausal women (5th–95th percentile; reference ranges provided by Bioclinica). *Nominal Wilcoxon test *p* value <5%. CRP: C-reactive protein; CXCL13: chemokine (C-X-C motif) ligand 13; Lp(a): lipoprotein (a); MMP‑3: matrix metalloproteinase-3; OC: osteocalcin; OPG: osteoprotegerin; P1NP: procollagen type 1 *N*-terminal propeptide; Q: quartile; q2w: every 2 weeks; RANKL: receptor activator of nuclear factor-κB ligand; SAA: serum amyloid A; sICAM-1: soluble intercellular adhesion molecule-1; TIBC: total iron-binding capacity.

**Table S4** Absolute change from baseline in biomarker concentrations through week 24

| **Median absolute change from baseline (Q1, Q3)** | | **Week 2** | **Week 12** | **Week 24** |
| --- | --- | --- | --- | --- |
| SAA, ng/mL | Adalimumab 40 mg q2w | — | –2442.7  (–22330.7, 1930.9) | –476.4  (–19654.3, 3342.1) |
|  | Sarilumab  200 mg q2w | — | –9066.5  (–80132.7, –1902.8) | –9604.7  (–79255.8, –1398.0) |
| CRP, mg/L | Adalimumab 40 mg q2w | — | –1.3  (–13.7, 1.3) | –1.3  (–13.7, 3.5) |
|  | Sarilumab  200 mg q2w | — | –6.8  (–22.9, –1.6) | –6.6  (–22.8, –1.3) |
| Lp(a), mg/L | Adalimumab 40 mg q2w | — | –2.0  (–54.0, 28.0) | –4.5  (–40.0, 28.0) |
|  | Sarilumab  200 mg q2w | — | –59.0  (–134.0, –13.0) | –60.3  (–157.0, –21.0) |
| MMP-3, ng/mL | Adalimumab 40 mg q2w | — | — | –5.6  (–23.6, 6.3) |
|  | Sarilumab  200 mg q2w | — | — | –6.8  (–33.1, 1.4) |
| Total RANKL, pmol/L | Adalimumab 40 mg q2w | 15.9  (–7.2, 129.1) | — | 31.5  (–93.5, 223.2) |
|  | Sarilumab  200 mg q2w | –10.5  (–69.5, 31.0) | — | –76.8  (–438.5, 20.4) |
| P1NP, ng/mL | Adalimumab 40 mg q2w | — | — | 2.0  (–5.5, 13.5) |
|  | Sarilumab  200 mg q2w | — | — | 8.6  (–0.8, 18.7) |
| OPG, pmol/L | Adalimumab 40 mg q2w | –0.2 (–0.7, 0.3) | — | 0.2  (–0.6, 0.9) |
|  | Sarilumab  200 mg q2w | 0.1  (–0.5, 0.7) | — | 0.1  (–0.7, 0.7) |
| OC, ng/mL | Adalimumab 40 mg q2w | — | — | 0.9  (–1.7, 5.1) |
|  | Sarilumab  200 mg q2w | — | — | 2.4  (–1.4, 5.7) |
| CXCL13, pg/mL | Adalimumab 40 mg q2w | –45.4  (–81.7, –22.9) | — | –30.5  (–65.7, –1.5) |
|  | Sarilumab  200 mg q2w | –12.8  (–41.0, 5.6) | — | –35.7  (–80.5, –4.5) |
| sICAM-1 | Adalimumab 40 mg q2w | –23.2  (–37.8, –9.3) | — | –10.8  (–41.7, 14.4) |
|  | Sarilumab  200 mg q2w | –0.7  (–15.6, 12.8) | — | –11.0  (–36.7, 1.9) |
| Iron, µmol/L | Adalimumab 40 mg q2w | 1.4  (–0.6, 4.5) | — | — |
|  | Sarilumab  200 mg q2w | 3.7  (0.5, 8.3) | — | — |
| Ferritin, ng/mL | Adalimumab 40 mg q2w | –8.5  (–31.7, 0.5) | — | — |
|  | Sarilumab  200 mg q2w | –7.7  (–26.4, 0.1) | — | — |
| TIBC, µg/dL | Adalimumab 40 mg q2w | 9.0  (–2.0, 22.0) | — | — |
|  | Sarilumab  200 mg q2w | 20.0  (5.0, 35.0) | — | — |
| Hepcidin, ng/mL | Adalimumab 40 mg q2w | –5.8  (–19.1, –0.1) | — | — |
|  | Sarilumab  200 mg q2w | –3.3  (–16.3, 0.4) | — | — |

Sample size in the overall biomarker population: adalimumab 40 mg q2w: *n* = 154, sarilumab 200 mg q2w: *n* = 153. CRP: C-reactive protein; CXCL13: chemokine (C-X-C motif) ligand 13; Lp(a): lipoprotein (a); MMP‑3: matrix metalloproteinase-3; OC: osteocalcin; OPG: osteoprotegerin; P1NP: procollagen type 1 *N*-terminal propeptide; Q: quartile; q2w: every 2 weeks; RANKL: receptor activator of nuclear factor-κB ligand; SAA: serum amyloid A; sICAM-1: soluble intercellular adhesion molecule-1; TIBC: total iron-binding capacity.

| **Table S5** Percentage of patients with CRP ≤10 mg/L and ≤3 mg/L at weeks 12 and 24 (overall safety population) | | | | |
| --- | --- | --- | --- | --- |
|  | **Week 12** | | **Week 24** | |
|  | **Adalimumab  40 mg q2w (*n* = 185)** | **Sarilumab  200 mg q2w (*n* = 184)** | **Adalimumab  40 mg q2w (*n* = 154)** | **Sarilumab  200 mg q2w (*n* = 153)** |
| CRP ≤3 mg/L, n (%) | 64 (38.1) | 148 (89.2) | 53 (34.0) | 149 (90.9) |
| CRP ≤10 mg/L, n (%) | 110 (65.5) | 156 (94.0) | 100 (64.1) | 157 (95.7) |

CRP: C-reactive protein; q2w: every 2 weeks

| **Table S6** Percentage of patients with anaemia at weeks 2 and 24 (overall safety population) | | |
| --- | --- | --- |
|  | **Adalimumab  40 mg q2w (*n* = 185)** | **Sarilumab  200 mg q2w (*n* = 184)** |
| Baseline | 25.4% | 24.5% |
| Week 2 | 21.1% | 19.6% |
| Week 24 | 16.2% | 10.9% |

Anaemia defined by World Health Organization criteria (haemoglobin <12 g/dL [women]/<13 g/dL [men]). q2w: every 2 weeks

**Table S7** Treatment-by-tertile biomarker interactions for efficacy endpoints at week 24 analysed by baseline biomarker in tertiles

| **Efficacy endpoint at week 24** | **Biomarker at baseline** | | | | | | | | | | | | | |
| --- | --- | --- | --- | --- | --- | --- | --- | --- | --- | --- | --- | --- | --- | --- |
|  | SAA | | CRP | | MMP-3 | | OPG | | OC | | CXCL13 | | Hepcidin | |
|  | M/L | H/L | M/L | H/L | M/L | H/L | M/L | H/L | M/L | H/L | M/L | H/L | M/L | H/L |
| ACR20 | NS | **0.015** | NS | **0.039** | NS | **0.013** | NS | NS | **0.031** | NS | NS | **0.003** | NS | **0.021** |
| ACR50 | NS | **0.004** | NS | NS | NS | NS | NS | NS | NS | NS | NS | NS | NS | NS |
| ACR70 | NS | **0.008** | NS | NS | NS | NS | **0.032** | NS | NS | NS | NS | NS | NS | NS |
| DAS28-ESR <2.6 | NS | NS | NS | NS | NS | NS | NS | NS | NS | NS | NS | NS | NS | NS |
| DAS28-ESR <3.2 | **0.004** | NS | NS | NS | NS | NS | NS | NS | NS | NS | NS | NS | NS | NS |
| DAS28-CRP <2.6 | NS | **0.041** | NS | NS | NS | NS | NS | NS | NS | NS | NS | NS | NS | NS |
| DAS28-CRP <3.2 | NS | **0.044** | NS | **0.049** | NS | **0.014** | NS | NS | NS | NS | NS | NS | NS | NS |

M/L: Nominal treatment-by-biomarker interaction *p*-value for Medium vs. Low tertile; H/L: Nominal treatment-by-biomarker interaction *p* value for High vs. Low tertile. ACR20/50/70: American College of Rheumatology 20/50/70% responses; CRP: C-reactive protein; CXCL13: chemokine (C-X-C motif) ligand 13; DAS28 CRP: Disease Activity Score (28 joints) C-reactive protein; DAS28-ESR: Disease Activity Score (28 joints) erythrocyte sedimentation rate; MMP-3: matrix metalloproteinase 3; NS: not significant at 5%; OC: osteocalcin; OPG: osteoprotegerin; SAA: serum amyloid A.

**Table S8** Treatment-by-tertile biomarker interactions for PROs at week 24 analysed by baseline biomarker in tertiles

| **Change from baseline in PROs at Week 24** | **Biomarker at baseline** | | | | | | | | | | | | | | | | | |
| --- | --- | --- | --- | --- | --- | --- | --- | --- | --- | --- | --- | --- | --- | --- | --- | --- | --- | --- |
|  | **SAA** | | **CRP** | | **MMP-3** | | **OC** | | **CXCL13** | | **Hepcidin** | | **s-ICAM1** | | **Iron** | | **Ferritin** | |
|  | M/L | H/L | M/L | H/L | M/L | H/L | M/L | H/L | M/L | H/L | M/L | H/L | M/L | H/L | M/L | H/L | M/L | H/L |
| Patient global VAS | NS | **0.011** | **0.002** | **0.040** | NS | **0.010** | NS | NS | NS | NS | NS | **0.009** | NS | NS | NS | NS | NS | NS |
| HAQ-DI | **0.035** | **<0.001** | NS | **0.005** | NS | **<0.001** | NS | NS | **0.032** | **0.004** | NS | NS | NS | NS | NS | **0.032** | NS | NS |
| Pain VAS | NS | **0.002** | **0.021** | **0.029** | **0.047** | **0.002** | NS | NS | NS | NS | **0.010** | **0.002** | NS | NS | NS | NS | NS | NS |
| SF-36 – PCS score | NS | **<0.001** | **0.009** | **0.016** | NS | **0.026** | NS | NS | NS | **0.031** | NS | NS | NS | NS | NS | NS | NS | NS |
| SF-36 – MCS score | NS | NS | NS | NS | NS | NS | NS | NS | NS | NS | **0.030** | **0.023** | NS | NS | NS | NS | NS | **0.050** |
| SF-36 – PF domain | NS | **0.003** | NS | NS | NS | **0.036** | NS | NS | NS | **0.003** | NS | NS | NS | NS | NS | NS | NS | NS |
| SF-36 – BP domain | NS | NS | NS | NS | NS | NS | **0.037** | NS | NS | NS | **0.002** | **0.016** | NS | NS | NS | NS | NS | NS |
| SF-36 – VT domain | NS | NS | NS | NS | NS | NS | NS | NS | NS | NS | **0.043** | **0.005** | NS | NS | NS | NS | NS | NS |
| SF-36 – RE domain | NS | NS | **0.049** | NS | NS | NS | NS | NS | NS | NS | NS | NS | NS | **0.030** | NS | NS | NS | NS |
| SF-36 –MH domain | NS | NS | NS | NS | NS | NS | NS | NS | NS | NS | **0.004** | **0.004** | NS | NS | NS | NS | **0.047** | NS |
| Morning stiffness VAS | NS | **0.004** | **0.002** | **0.017** | NS | **<0.001** | NS | NS | NS | NS | **0.029** | **<0.001** | NS | NS | NS | NS | NS | NS |
| RAID score | NS | **0.017** | **0.045** | NS | NS | **0.020** | NS | NS | NS | NS | NS | **0.009** | **0.032** | NS | NS | NS | NS | NS |

M/L: Nominal treatment-by-biomarker interaction *p* value for Medium vs. Low tertile; H/L: Nominal treatment-by-biomarker interaction *p* value for High vs. Low tertile. BP: bodily pain; CRP: C-reactive protein; CXCL13: chemokine (C-X-C motif) ligand 13; FACIT: Functional Assessment of Chronic Illness Therapy; HAQ-DI: Health Assessment Questionnaire-Disability Index; Lp(a): lipoprotein (a); MCS: mental component summary; MH: mental health; MMP-3: matrix metalloproteinase-3; NS: not significant at 5%; OC: osteocalcin; OPG: osteoprotegerin; P1NP: procollagen type 1 *N*-terminal propeptide; PCS: physical component summary; PF: physical functioning; RAID: rheumatoid arthritis impact of disease; RANKL: receptor activator of nuclear factor-κB ligand; RE: role-emotional; SAA: serum amyloid A; SF-36: Medical Outcomes Study Short-Form (36-item) Health Survey; sICAM-1: soluble intercellular adhesion molecule-1; TIBC: total iron-binding capacity; VAS: visual analogue scale; VT: vitality.

**Fig. S1** Correlation matrix for baseline biomarkers and haematology parameters


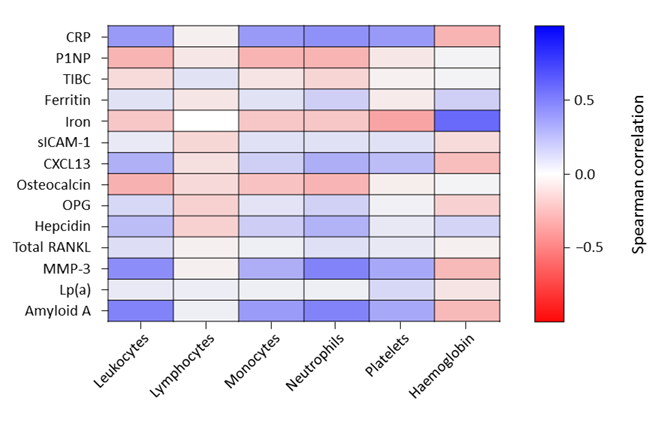


CRP: C-reactive protein; CXCL13: chemokine (C-X-C motif) ligand 13; Lp(a): lipoprotein (a); MMP-3: matrix metalloproteinase-3; OPG: osteoprotegerin; P1NP: procollagen type 1 *N*-terminal propeptide; RANKL: receptor activator of nuclear factor-κB ligand; sICAM-1: soluble intercellular adhesion molecule-1; TIBC: total iron-binding capacity

**Fig. S2** Median percentage changes from baseline in (A) CXCL13 and (B) sICAM-1 through week 24

**
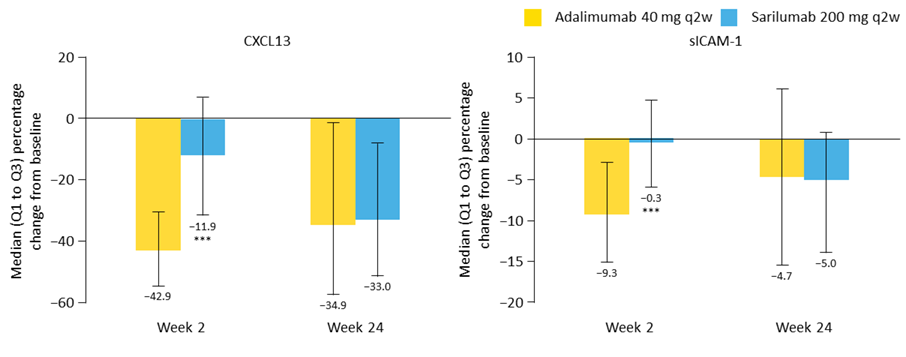
**

***Adjusted *p* < 0.0001 vs. adalimumab (Benjamini–Hochberg procedure). CXCL13: chemokine (C-X-C motif) ligand 13; Q: quartile; q2w: every 2 weeks; sICAM-1: soluble intercellular adhesion molecule-1

**Fig. S3** Median percentage changes from baseline in biomarkers of anaemia of chronic disease 2 weeks post-treatment

**
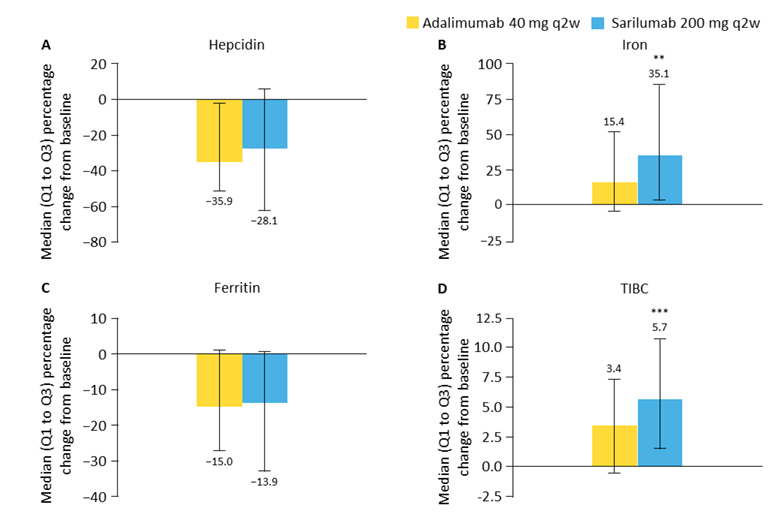
**

Median percentage changes from baseline in (A) hepcidin, (B) iron, (C) ferritin, and (D) TIBC at week 2 post-treatment. ***Adjusted *p* < 0.0001 vs. adalimumab (Benjamini–Hochberg procedure). **Adjusted *p* < 0.01 vs. adalimumab (Benjamini–Hochberg procedure). Q: quartile; q2w: every 2 weeks; TIBC: total iron-binding capacity

**Fig. S4** ACR50 responses at week 24 and corresponding ORs with differential combinations of CXCL13 and sICAM-1

**
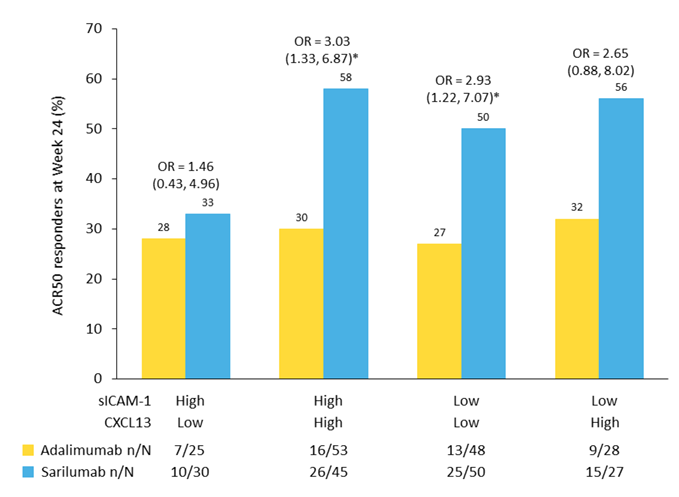
**

*Nominal *p* < 0.05. ORs presented with 95% CIs. ACR50: American College of Rheumatology 50% improvement criteria; CI: confidence interval; CXCL13: chemokine (C-X-C motif) ligand 13; OR: odds ratio; q2w: every 2 weeks; sICAM-1: soluble intercellular adhesion molecule-1.
